# Extracellular DNA of slow growers of mycobacteria and its contribution to biofilm formation and drug tolerance

**DOI:** 10.1038/s41598-021-90156-z

**Published:** 2021-05-26

**Authors:** Aleksandr Ilinov, Akihito Nishiyama, Hiroki Namba, Yukari Fukushima, Hayato Takihara, Chie Nakajima, Anna Savitskaya, Gebremichal Gebretsadik, Mariko Hakamata, Yuriko Ozeki, Yoshitaka Tateishi, Shujiro Okuda, Yasuhiko Suzuki, Yuri S. Vinnik, Sohkichi Matsumoto

**Affiliations:** 1 Department of Bacteriology, Niigata University School of Medicine, 1-757, Asahimachi-Dori, Chuo-ku, Niigata, Niigata 951-9510 Japan; 2 Department of General Surgery Named Professor M.I. Gulman, Professor V.F. Voino-Yasenetsky Krasnoyarsk State Medical University, 1, P. Zheleznyaka str., Krasnoyarsk, Russian Federation 660022; 3 Division of Bioresources, Hokkaido University Research Center for Zoonosis Control, Sapporo, 011-0020 Japan; 4 International Collaboration Unit, Hokkaido University Research Center for Zoonosis Control, Sapporo, 011-0020 Japan; 5 Department of Advanced Pharmaceutics, Faculty of Veterinary Medicine, Hokkaido University, Sapporo, 060-0818 Japan; 6 Division of Bioinformatics, Niigata University School of Medicine, 1-757, Asahimachi-Dori, Chuo-ku, Niigata, Niigata 951-9510 Japan; 7 Shemyakin-Ovchinnikov Institute of Bioorganic Chemistry, Russian Academy of Sciences, Moscow, Russian Federation 117997; 8 Department of Respiratory Medicine and Infectious Disease, Niigata Graduate School of Medical and Dental Sciences, 1-757, Asahimachi-Dori, Chuo-ku, Niigata, Niigata 951-9510 Japan; 9 Laboratory of Tuberculosis, Institute of Tropical Disease, Universitas Airlangga, Kampus C Jl. Mulyorejo, Surabaya, 60115 Indonesia

**Keywords:** Microbiology, Infectious diseases, Respiratory tract diseases

## Abstract

DNA is basically an intracellular molecule that stores genetic information and carries instructions for growth and reproduction in all cellular organisms. However, in some bacteria, DNA has additional roles outside the cells as extracellular DNA (eDNA), which is an essential component of biofilm formation and hence antibiotic tolerance. Mycobacteria include life-threating human pathogens, most of which are slow growers. However, little is known about the nature of pathogenic mycobacteria’s eDNA. Here we found that eDNA is present in slow-growing mycobacterial pathogens, such as *Mycobacterium tuberculosis*, *M. intracellulare*, and *M. avium* at exponential growth phase. In contrast, eDNA is little in all tested rapid-growing mycobacteria. The physiological impact of disrupted eDNA on slow-growing mycobacteria include reduced pellicle formation, floating biofilm, and enhanced susceptibility to isoniazid and amikacin. Isolation and sequencing of eDNA revealed that it is identical to the genomic DNA in *M. tuberculosis* and *M. intracellulare*. In contrast, accumulation of phage DNA in eDNA of *M. avium*, suggests that the DNA released differs among mycobacterial species. Our data show important functions of eDNA necessary for biofilm formation and drug tolerance in slow-growing mycobacteria.

## Introduction

DNA is an essential molecule in all cellular organisms which stores genetic information required for development, growth, reproduction, and evolution. It has also been shown that DNA has an important role outside the cell, and it is termed as extracellular DNA (eDNA)^1–4^. Initially eDNA was detected in the culture supernatant of the biofilm forming bacterial species*, Pseudomonas aeruginosa*^3–6^. The role of eDNA is enormous in some bacterial life: eDNA is a major component necessary for genome repair, nutrition and horizontal gene transfer, transformation^2,5,7^, and biofilm formation^3,4^. eDNA has long been known as one of the most abundant molecules in mucous biofilm formed by different microorganisms such as halophiles^8^. Biofilm formation give bacteria a growth advantage in that it helps them become more tolerant to harsh conditions, resistant to antibiotics, persist in chronic disease and can even reduce efficacy of vaccines^9–15^. Biofilm formation thus causes health-threating impact on pathogenic bacterial infection.

Mycobacteria include life-threating human pathogens. Currently, *Mycobacterium tuberculosis* var. *tuberculosis* (*Mtb*) kills more people than any other single infectious agents. In 2018 alone 1.5 million people died from tuberculosis^16^. Nontuberculous mycobacterial (NTM) diseases are also increasing, especially in developed countries^16^. It is known that more than six months of chemotherapy is required to treat tuberculosis. Failure to complete such long-term chemotherapy frequently occurs without adequate control of patients, which in turn leads to development of drug resistant tuberculosis. As for NTM diseases, more than one year of chemotherapy is required and most NTM diseases are resistant to treatment, even when in vitro drug-sensitivity test show effectiveness. These suggest that biofilm formation of mycobacterial pathogens induce phenotypic drug tolerance that requires long-term chemotherapy for curing.

Mycobacteria, such as *Mtb, Mycobacterium avium, Mycobacterium marinum, Mycobacterium ulcerans,* and *Mycolicibacterium smegmatis* are known to form many types of biofilms^17–24^. The pellicle is a kind of biofilm at the air–liquid interface and one of the standard models of analysis of biofilm formation in mycobacteria. Generally, biofilm formation in bacteria is induced by oligotrophy that reflects harsh conditions. In contrast, formation of mycobacterial pellicle is enhanced by high carbon dioxide and low oxygen tensions, and eutrophy rather than oligotrophy^19,20,25–28^. These characteristics might be reflected in biofilm formation of *Mtb* in lungs with a high density of carbon dioxide and inside oxygen-depleted tuberculous granuloma. Biofilm formation by mycobacteria can be related to long-term habitat of NTM in bathrooms which is a major infectious source in human environment^29^. Components of mycobacterial biofilms are also unique, in part because in addition to exopolysaccharides (EPS) matrix contain free mycolic acids, glycopeptidolipids, and other lipid-containing molecules^27^. Despite the presence of such unique components in mycobacterial biofilms, eDNA is found in mycobacteria biofilm as seen in other bacteria.

eDNA can be released by different mechanism. The most common mechanism is bacterial lysis, although lytic-independent mechanisms (eDNA releasing independently of cell lysis) also exist^4,6,7,30^. It was first found in *P. aeruginosa* and later seen in the biofilm matrix of many other bacterial genera, including *Enterococcus faecalis, Bacillus cereus, M. avium* subsp. *Hominissuis*^2,3,6,24,30^. pH-dependent export of eDNA was observed in *M. avium* and FtsK/SpoIIIE were identified its responsible molecules. Condition of appearance of eDNA depends on the environment: attachment surface, nutrients, mechanical challenges, and stress conditions etc.^31^.

Understanding factors mediating biofilm formation of pathogens is an important step for disease control but the nature of eDNA is largely unknown in mycobacteria. In this study, we evaluated the physiological functions of mycobacterial eDNA.

## Results

### Different level of eDNA between slow and rapid growers of mycobacteria growing in 7H9-ADC broth

We first assessed the level of eDNA among mycobacterial species by employing slow-growing mycobacteria, such as *Mycobacterium tuberculosis* var. BCG Tokyo 172 (BCG), *Mycobacterium intracellulare* 13950, *Mycobacterium tuberculosis* H37Rv (*Mtb*), and *Mycobacterium avium* subsp. *hominissuis* 104 and fast-growing mycobacteria, such as *Mycolicibacterium fortuitum* subsp. *fortuitum* ATCC 6841*, Mycolicibacterium phlei* 5865^T^ (ATCC 19249), *Mycolicibacterium smegmatis* mc^2^ 155*,* and *Mycobacteroides abscessus* subsp. *abscessus* ATCC 14472. Bacteria were cultured in the 7H9-ADC medium until exponential growth phase with optical density value 600 nm (OD_600_) of 0.2. Then, we stained the bacteria with both SYTOX Green (SG) and calcein violet with an acetoxy-methyl ester group (CV-AM). SG stains eDNA only, because it cannot penetrate an intact cell membrane and cell wall^32^. CV-AM is an indicator of the presence of esterase, which is a marker of viable bacteria^33^.

**Figure 1:**
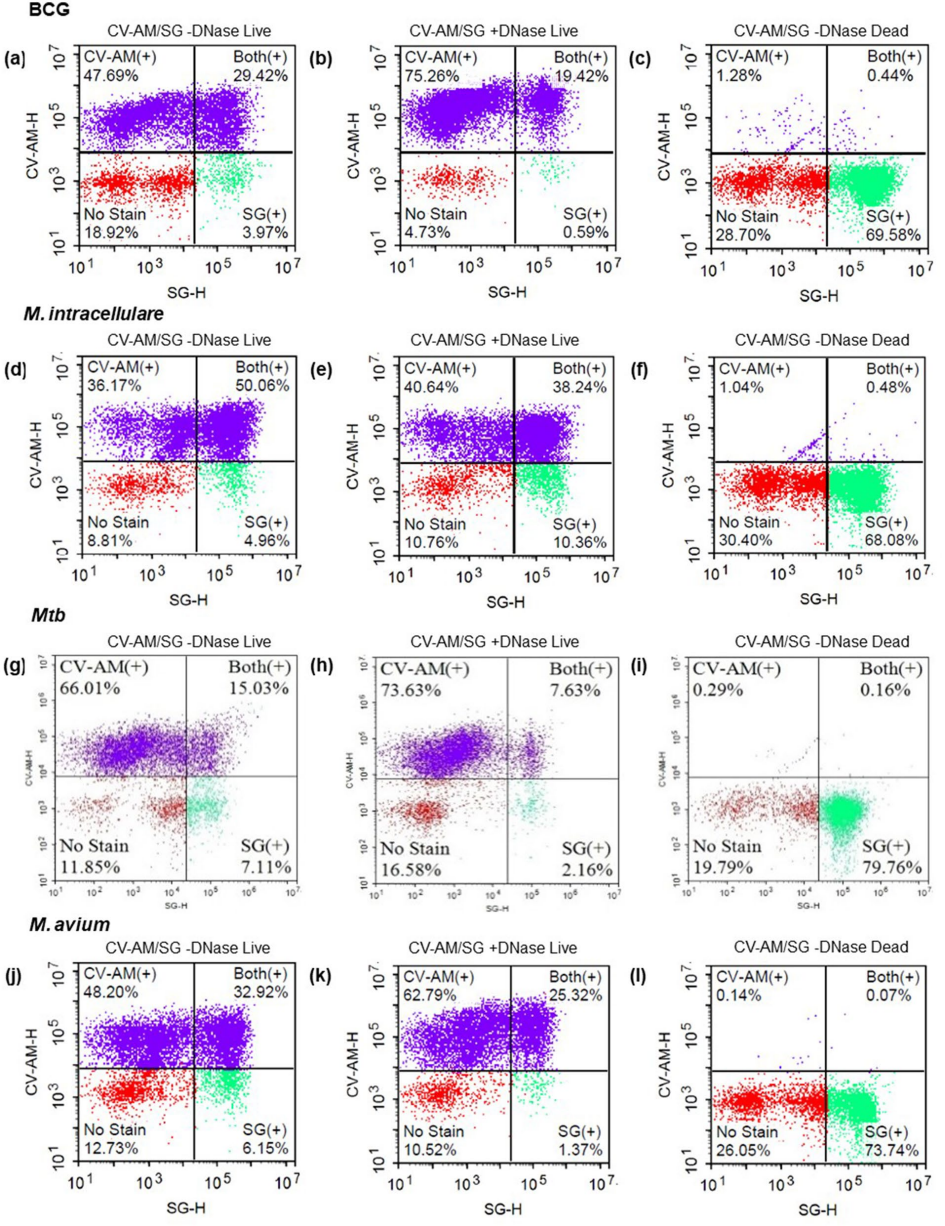
FACS analysis of extracellular DNA (eDNA) on BCG, *M. intracellulare*, *Mtb,* and *M. avium* in 7H9-ADC broth. Representative NovoCyte Flow Cytometer plots of BCG (**a**, **b**, and **c**), *M. intracellulare* (**d**, **e**, and **f**), *Mtb* (**g**, **h**, and **i**), and *M. avium* (**j**, **k**, and **l**) stained by calcein violet with an acetoxy-methyl ester group (CV-AM) typical of live cells and SYTOX Green (SG) stains DNA, respectively. Bacteria were treated with Benzonase Nuclease I (**b**, **e**, **h**, and **k**) or heated at 95 °C for 5 min (**c**, **f**, **i**, and **l**) before staining.

The percentage of SG and CV-AM double-positive BCG, *M. intracellulare*, *Mtb,* and *M. avium* were 29.42%, 50.06%, 15.03%, and 32.92%, respectively (Figs. 1a,d,g,j, 3). This data indicates the presence of DNA outside viable bacterial cells. As a negative control, we used heat killing bacteria, which showed double-positive result to under 0.5% and increased SG-single positive indicating that CV-AM does not stain dead bacteria (Figs. 1c,f,i,l, 3).

This unexpected higher percentage of double-stained slow-growing bacteria compared to a previous study^34^ forced us to think the portion of double-stained cells possess eDNA. To confirm this hypothesis, we treated bacteria with Benzonase Nuclease I (DNase I). We found a substantial decrease in double-positive bacteria such as BCG, *M. intracellulare*, *Mtb,* and *M. avium* which were 19.42%, 38.24%, 7.63%, and 25.32%, respectively (Figs. 1b,e,h,k, 3). These data suggest the presence of DNase I sensitive eDNA for every tested strain of slow-growing mycobacteria, although more than half of the portions were resistant to DNase I treatment, according to the percentage of double-stained cells slow-growing mycobacteria (Figs. 1b,e,h,k, 3).

**Figure 2:**
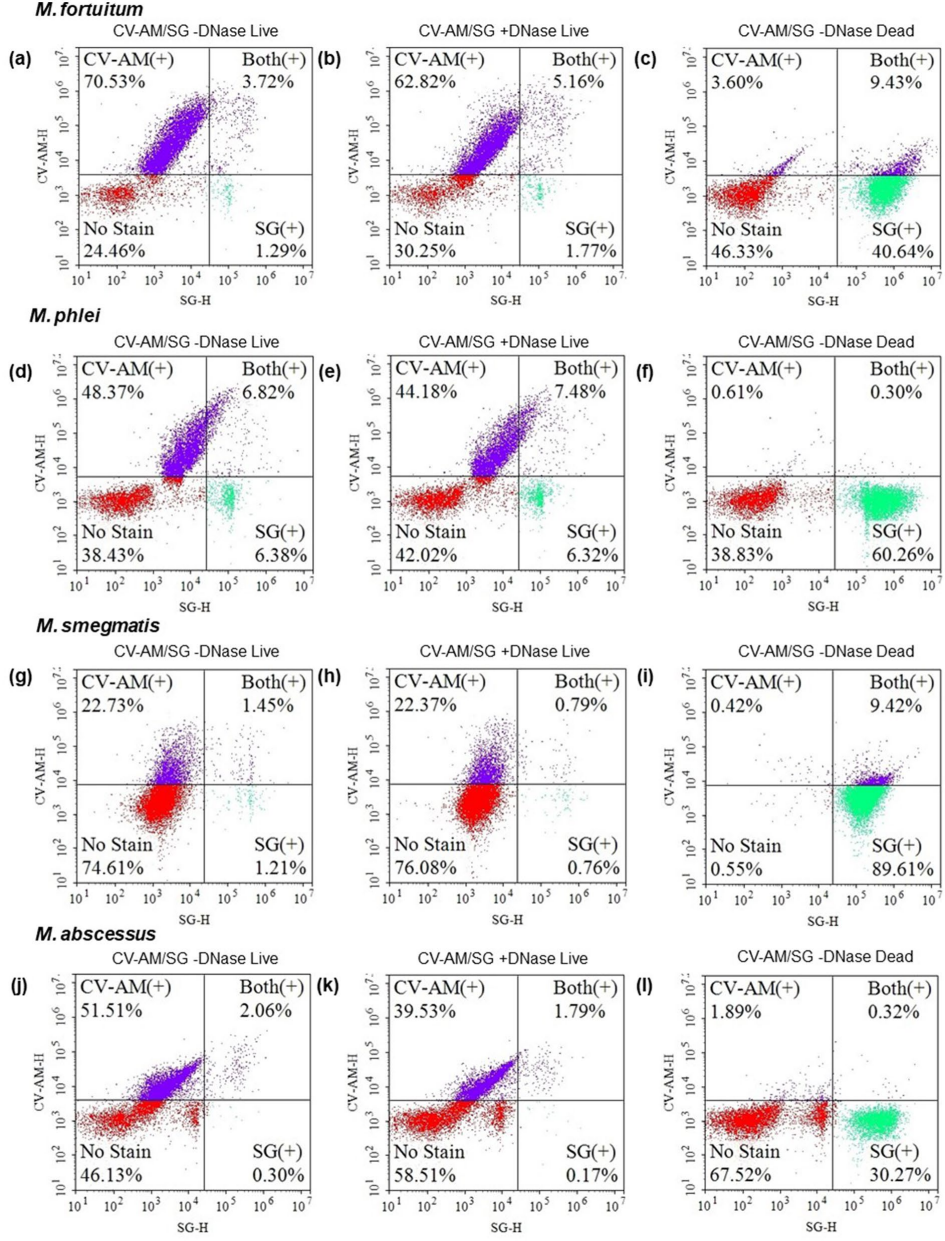
FACS analysis of extracellular DNA (eDNA) on *M. fortuitum, M. phlei, M. smegmatis, and M. abscessus* in 7H9-ADC broth. Representative NovoCyte Flow Cytometer plots of *M. fortuitum* (**a**, **b**, and **c**), *M. phlei* (**d**, **e**, and **f**), *M. smegmatis* (**g**, **h** and **i**), and *M. abscessus* (**j**, **k**, and **l**) stained by calcein violet with an acetoxy-methyl ester group (CV-AM) typical of live cells and SYTOX Green (SG) stains DNA, respectively. Bacteria were treated with Benzonase Nuclease I (**b**, **e**, **h**, and **k**) or heated at 95 °C for 5 min (**c**, **f**, **i**, and **l**) before staining.

**Figure 3:**
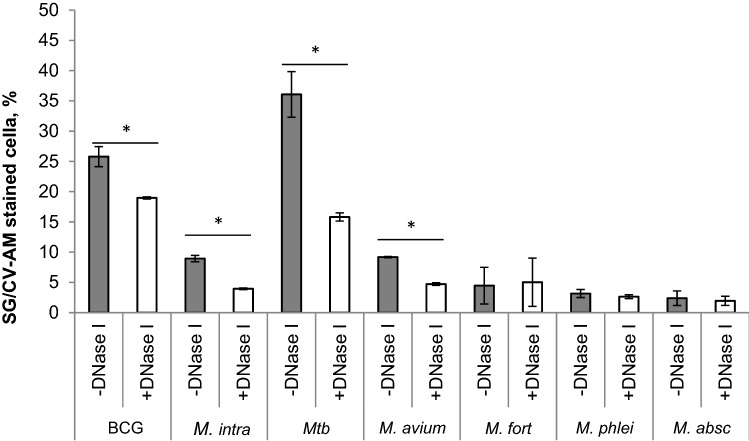
Comparative analysis of eDNA of slow-growing and fast-growing mycobacteria. Percentage of double-stained cells DNase I untreated (− DNase I) or treated (+ DNase I) slow-growing mycobacteria—BCG, *M. intracellulare*, *Mtb*, or *M. avium,* and fast-growing mycobacteria—*M. fortuitum, M. phlei, M. abscessus* stained with CV-AM/SG. Mean ± SD (n = 3). *p < 0.05, compared to DNase I untreated (− DNase I) and treated (+ DNase I) cells.

In contrast, the amount of double-stained fast-growing mycobacteria (*M. fortuitum, M. phlei*, *M. smegmatis*, and *M. abscessus*) were low and did not exceed 7%, although *M. smegmatis* was relatively resistant to CV-AM staining. Independent triplicated estimation of % of double-positive cells of *M. fortuitum, M. phlei*, and *M. abscessus* are 3.72 ± 0.9, 6.82 ± 0.3, and 2.06 ± 0.9, respectively. These small portions of double-stained population were constantly low when bacteria were treated with DNase I (Figs. 2, 3), showing little eDNA on rapid-growing mycobacteria.

The amounts of eDNA, triplicate evaluation, with or without DNase I treatment showed in Fig. 3. The data demonstrate that slow growers of mycobacteria are eDNA positive and the percentage of double-positive cells are varied from 15% up to 50%. On the other hand, the percentage of double-positive cells in rapid-growing bacteria are varied from 1 to 6%, which we define as eDNA negative.

Taken all together, these data indicate that the presence of eDNA is remarkable for slow-growing mycobacteria but not for fast-growing ones when mycobacteria are cultured in standard eutrophic media (7H9 supplemented with ADC).

### The impact of eDNA on pellicle formation

It has been shown that eDNA promotes a biofilm formation in *P. aeruginosa, Enterococcus spp.* and others. Mycobacterial species including pathogenic and non-pathogenic organisms form biofilms in various environmental reservoirs; we thought that eDNA would contribute to biofilm formation in slow-growing mycobacteria.

**Figure 4:**
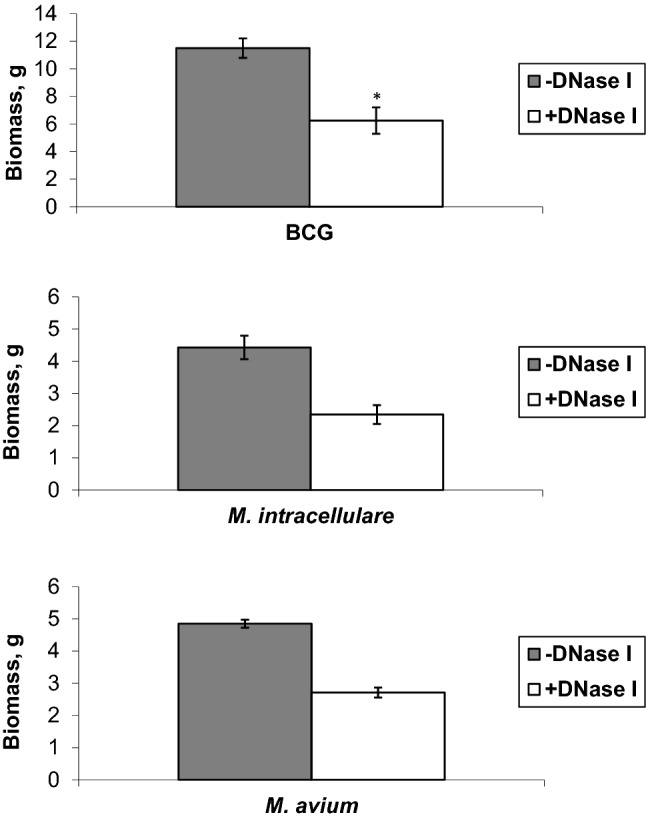
Biomass of mycobacterial pellicles treated with DNase I or not. Five weeks after incubation of BCG (**a**)*, M. intracellulare* (**b**), and *M. avium* (**c**) in Sauton medium with or without the addition of DNase I and wet weights of pellicle were measured. Mean ± SD (n = 4). *p < 0.05, compared to a biomass of DNase I untreated BCG (− DNase I).

In order to address this, we cultured BCG, *M. intracellulare*, and *M. avium* in Sauton medium, a standard non-detergent media and good for pellicle formation in mycobacterial growth, with or without the addition of DNase I every week. Two to three weeks later, these bacteria formed thin pellicle biofilms on the surface of the media and prolonged incubation lead to the formation of a thick floating biofilm. After five weeks we collected the bacterial biomass and passed through a 0.45 µm filter to remove water and measured the weight of the biomass. We observed a reduction in biomass formation for all tested mycobacterial species after incubation with DNase I (Fig. 4). BCG, *M. intracellulare, M. avium* had above twofold difference between treated and untreated DNase I samples. These data showed that the disruption of eDNA led to a decrease of biomass. Thus, eDNA has a structural role and promotes formation of biofilms of slow growers of mycobacteria.

### eDNA aids in tolerance of mycobacteria to certain drugs

Biofilm formation has been shown to be advantageous in bacterial growth especially when it comes to tolerance to antibiotics. However, due to solid clumped mycobacterial biofilm, measuring the exact number of drug-tolerant mycobacteria in biofilm is difficult. Thus, we examined whether eDNA contributes to mycobacterial tolerance to drugs.

BCG, *M. intracellulare,* and *M. avium* were pre-cultured in 7H9-ADC medium till OD_600_ of 0.1 and diluted to OD_600_ of 0.001, and further cultured in the presence or absence of DNase I for 72 h. Then, incubated with isoniazid (INH), rifampicin (RMP), amikacin (AMK), and clarithromycin (CLA) for 6 and 24 h, and followed by a subsequent assessment of viability by counting the colony forming units (CFUs).

**Figure 5:**
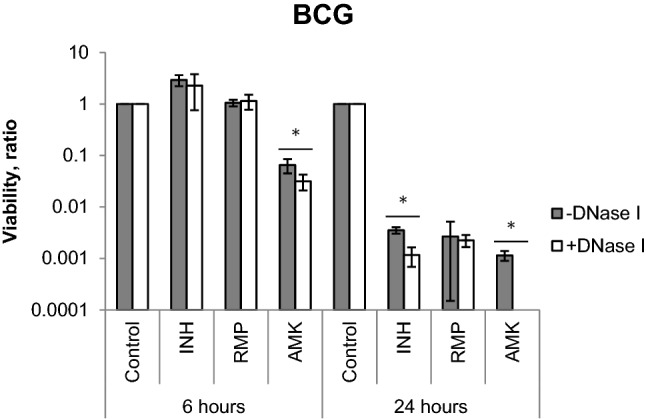
Differential effect of DNase I treatment BCG drug susceptibility. BCG was untreated (− DNase I) or treated (+ DNase I) at 37 °C for 72 h and further incubated with INH, RMP, AMK or not, for 6 and 24 h. CFU/ml was determined and normalized with that of control samples. The average viability ratio of each sample (mean ± SD, n = 3) is indicated. *p < 0.05.

Data are presented as a percentage of viable cells of BCG (Fig. 5), *M. intracellulare,* and *M. avium* (Supplementary Figure S1) relative to cells, untreated with DNase I. We found that DNase I treatment leads to decrease BCG viability (data not shown). DNase I treatment increased the efficacy of AMK and INH but not RMP. Significant reduction of CFUs was observed when BCG was treated with AMK for both 6 and 24 h, and with INH for 24 h (Fig. 5).

Treatment *M. intracellulare* and *M. avium* by DNase I only slightly reduced CFU in drug-free condition. A significant increasement of drug sensitivity was observed only in *M. avium* treated with CLA for 6 h as compared to control (Supplementary Fig. S1).

### Nature of eDNA

In order to know the nature of eDNA of mycobacteria, we purified both eDNA and genomic DNA (gDNA). eDNA was purified without the membrane disruption procedure. In order to confirm if our purification method of eDNA efficiently extract it, we employed RT-PCR-based method using propidium monoazide (PMAxx), which is a photo-reactive DNA-binding dye. This dye is impermeable to live cells, thus this dye only binds to eDNA of living bacteria and inhibits PCR by intercalation. Extraction of DNA by two different methods followed incubation with PMAxx showed that the amounts of PCR-amplification of 16S rRNA gene in gDNA fractions similar between PMAxx-treated and non-treated sample (1.5-fold difference). The amount of eDNA purified from the PMAxx-treated sample was 3.8-fold lower than that from non-treated sample (Supplementary Table S2). This data showed that the protocol is useful for efficient purification eDNA from mycobacteria.

**Figure 6:**
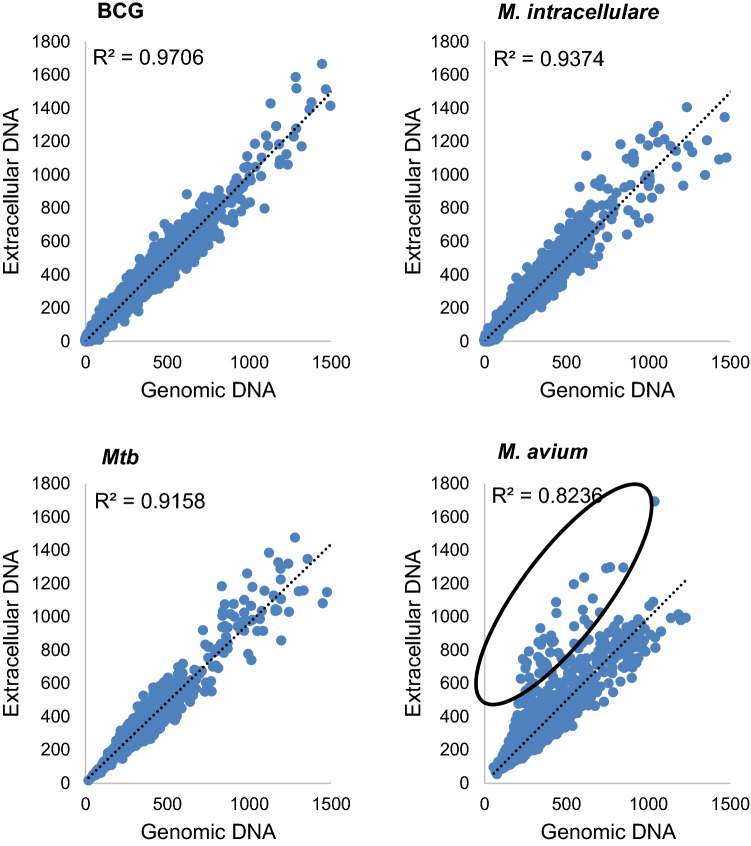
Whole genome sequencing comparison of gDNA and eDNA of BCG, *M. intracellulare, Mtb,* and *M. avium.* The relative abundance of gDNA and eDNA genes of BCG, *M. intracellulare*, *Mtb,* and *M. avium* were calculated. Each gene was plotted where the relative abundance of gDNA and eDNA correspond. The correlation of relative abundance of genes in each *Mycobacterium* species was 0.97, 0.94, 0.92, 0.82 for BCG, *M. intracellulare*, *Mtb,* and *M. avium*, respectively. The circle indicates accumulated genes in eDNA.

gDNA basically consist of genomic DNA, but we could not exclude the presence of some amount eDNA in BCG, *M. intracellulare*, *Mtb*, and *M. avium* as shown in Supplementary Fig. S2 and Supplementary Fig. S3. We sequenced both eDNA and gDNA using the MiSeq Illumina next-generation sequencer. Comparison of eDNA and gDNA sequences showed that most of the tested strains eDNA correspond to genome DNA. eDNA of BCG, *M. intracellulare,* and *Mtb* are identical with their gDNA, suggesting that the eDNA resulted from mycobacterial lysis or export of entire gDNA (Fig. 6).

**Figure 7:**
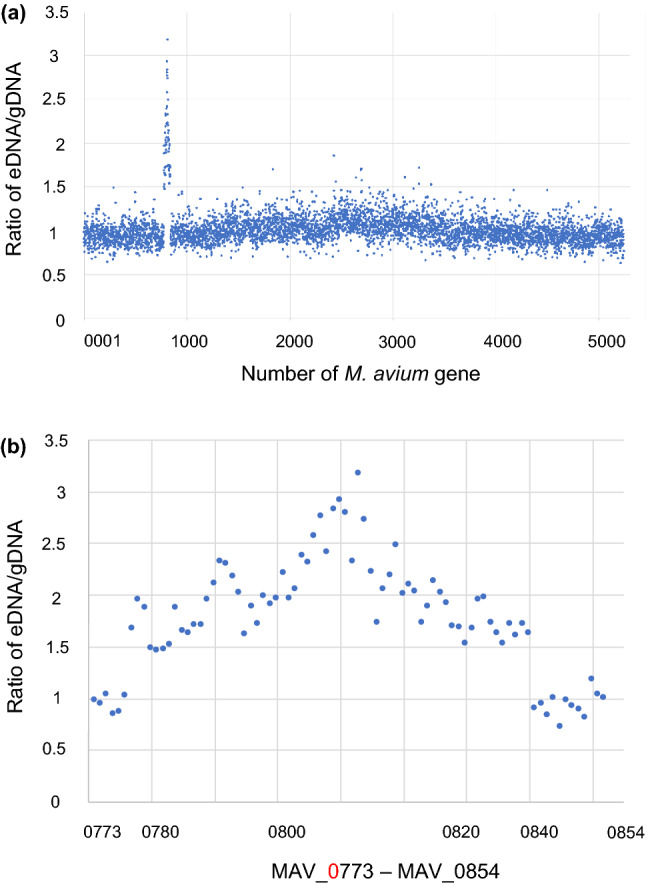
ratio of gDNA and eDNA of each gene in *M. avium* 104. (**a**) The abundance ratio of gDNA to eDNA in *M. avium* was calculated for each genes. (**b**) An expansion of (a) from MAV_0773 to MAV_0854.

Comparative analysis of eDNA and gDNA sequences *M. avium* revealed specific to the eDNA set of genes (Fig. 6). These sequences encoded MAV_0779 to 0842 which refers to genes of prophage phiMAV_1 (from MAV_0779 to MAV_0841)^35^ (Fig. 7, Supplementary Table S1). This suggests that phage DNA release is an additional way of eDNA construction in *M. avium* 104.

## Discussion

Biofilm formation is involved in resistance against chemotherapy in mycobacterial diseases, most of which are observed in slow-growers^17,20,23,27^. eDNA is a component of the bacterial biofilm^20,24,26^. Unexpectedly in this study, we found that slow growers of mycobacteria have a higher amount of eDNA in contrast to rapid growers of mycobacteria when they were planktonically cultured in 7H9-ADC media. Slow-growing pathogens, such as *M. tuberculosis* complex and MAC release detectable amount of eDNA which can afford pellicle formation, in turn lead to drug resistance. eDNA also contributed to drug tolerance even in planktonic exponential phase (Figs. 4,5). These data suggest a more prominent function of eDNA in slow-growing mycobacteria thus conferring a growth advantage and drug tolerance.

Rapid-growing mycobacteria have little eDNA at the exponential growth phase. This may suggest the importance of other molecules for biofilm development in rapid growers. Recht et al. showed that glycopeptidolipids and mycolic acid are essential for initial surface attachment during biofilm formation for *M. smegmatis*^28^. Thus, fast-growing bacteria probably may start to release eDNA at the later phase of biofilm formation for biofilm development^4,25^.

Treatment with antituberculosis drugs INH, RMP, and aminoglycosides AMK, CLA at two conditions (with and without DNase I at 6 and 24 h of treatment), showed heterogeneous results but revealed the contribution of eDNA to tolerance of mycobacteria to certain kinds of drugs, such as INH, AMK, and CLA. By contrast, DNase I treatment did not show any influence on the effect of RMP. This is maybe because RMP is highly lipophilic. Mycobacterial cell walls consist up to 60% of lipids. Mycobacterial lipids has many biological functions, including resistance of majority of mycobacteria species to most broad-spectrum antibiotics by low permeability rate. The lipophilic nature of RMP may allow to drug act independently from eDNA.

Treatment with AMK also showed a significant decrease of viability in contrast to control, and sensitivity to AMK was increased by the addition of DNase I (Fig. 5). Thus, the presence of eDNA somehow influences BCG’s viability. AMK is an aminoglycoside that irreversibly binds to the 30S subunit and inhibits protein synthesis in ribosomes. The possible reason for heterogeneous data of aminoglycosides effect on the viability of mycobacteria with/without DNase I treatment would be dissimilarity of the microenvironment such as humidity, mass transfer, pH, etc^27^.

To investigate if eDNA found in mycobacteria is genomic in origin or has an eDNA-specific sequence, extraction of eDNA was done, sequenced, and was directly compared to gDNA sequence. eDNA sequence identity with gDNA in BCG, *M. intracellulare*, and *Mtb* means that eDNA was likely occurred by bacterial lysis. Since eDNA was seen in slow growers of mycobacteria but was little in rapid growers (Fig. 1), it can be considered that a certain population of slow-growing mycobacteria die even at exponential growth phase. This can be explained by the bacterial death caused by entering the lysis cycle of intrinsic lysogenic bacteriophages. We actually observed accumulated phage DNA^36^ in the eDNA of *M. avium* 104, suggesting that some population of *M. avium* was lysed by phage lysis.

Lahiri A. and Lewin A. et al. performed a comparative analysis of genomic islands of *M. avium* strains. They found that a prophage composed of 45 genes from MAV_0786 to MAV_0830 is located in genomic island 3^36^. Our comparative analysis of eDNA and gDNA of *M. avium* determined a set of genes from MAV_0779 to MAV_0842 accumulates in eDNA (Supplementary Table S1). These suggest that bacteriophage annotated by Lahiri A. and Lewin A. et al. Actual release from *M. avium*. The discrepancy accumulated genes in eDNA and annotated phage genome may suggest that neighbour genes were packed in the phage particle when it releases or they are also phage genome unpredicted functions.

However, we did not identify phage DNA accumulation in the eDNA of other examined slow growers of mycobacteria. Another possibility of eDNA construction is frequent failure of replication, resulting to the death of mycobacteria. It is known that even in active disease state, a certain *Mtb* population shifts to a dormant phase, which necessitates the long treatment regimen for tuberculosis^32^. It is also known that a constant population of bacteria, past the stationary phase, lose viability and eventually die. Bacterial death releases gDNA to become eDNA which may support the survival of other live bacteria. Recent studies suggest that bacterial apoptosis and its mechanisms are important in biofilm development^10,12^. In case of nutrient limitation or antibiotics exposure, part of the bacterial population is sacrificed to provide a source of nutrients or eDNA that can be picked up viable cells and used for DNA repair, transformation, quorum sensing, etc.^9,15,17^. Such a proactive biological mechanism might be involved in the construction of eDNA in slow growers of mycobacteria during the planktonic exponential phase.

Using a high biofilm-forming strain of *M. avium* subsp. *hominissuis*, Rose et. al observed a significant effect eDNA on the development of biofilm and drug tolerance of *M. avium*^29^. Cloning of certain parts of the eDNA by polymerase chain reaction-based method and their sequencing showed that gDNA is the source of eDNA. Interestingly, a comparison of the quantity of eDNA in the biofilm matrix and the living number of bacteria by CFU in the same study didn’t show a significant decrease in CFU despite the increase in the amount of eDNA. This statement is an introduction to the other factor that is the source of eDNA in addition to bacterial death. There can be a mechanism that transfer large-size of DNA resemble bacterial conjugation^37^.

Similar to our study, other groups also showed that eDNA induces tolerance of bacterial biofilms. DNA release occurs during the drug-mediated killing of bacteria. Thus, powerful new drug, such as RMP (Fig. 5) that sterilizes mycobacteria covered by eDNA is required for controlling mycobacterial diseases. This study provides basic knowledge for the development of control strategies against intractable mycobacterial diseases.

## Methods

### Bacterial strains, growth, and reagents

All mycobacteria strains were grown in Middlebrook 7H9 broth (BD, Franklin Lakes, NJ) supplemented with 0.2% (v/v) glycerol, 0.05% (v/v) Tween 80 (MP Biomedicals, Santa Ana, CA), and 10% ADC enrichment (5% bovine serum albumin [Wako Pure Chemical Industries, Osaka, Japan], 0.81% NaCl, and 2% D-glucose) (7H9-ADC broth) or on Mycobacteria 7H11 agar (BD) supplemented with 0.5% (v/v) glycerol and 10% OADC enrichment (ADC enrichment supplemented with 0.06% [v/v] oleic acid) (7H11-OADC agar). For biofilm formation experiment mycobacteria strains were grown on liquid.

Hygromycin B (HYG), kanamycin sulfate (KAN), rifampicin (RMP), amikacin (AMK,) and clarithromycin (CLA) were purchased from Wako Pure Chemical Industries [Osaka, Japan]; and INH from SIGMA-ALDRICH [St. Louis, USA]. AMK stock solutions were prepared in water. Stock solutions of all other compounds were prepared in 100% dimethyl sulfoxide (DMSO) and then filter sterilized (pore size 0.45 µm). These components were frozen in aliquots at − 20 °C.

CV-AM and SG were purchased from Invitrogen (Life-Technologies Corporation, California, USA). CV-AM was dissolved in 250 µl (μl) of DMSO. SG was diluted from the manufacture’s 5 mM stock solution to a final concentration of 50 μM by DMSO. The dyes were stored at − 30 °C.

PMAxx dye was purchased from Cosmo Bio Co., LTD (Tokyo, Japan).

### DNase I treatment and staining with CV-AM and SG dyes

Bacterial culture was adjusted to an OD_600_ of 0.01. Each sample was divided into two Eppendorf tubes of 1 ml each and 1 μl (2 units) DNase I (SIGMA-ALDRICH) added to one of them. Samples were then incubated overnight at 37 °C with rotation of 4 rpm. One hundred μl was added to a fresh Eppendorf tube. Five μl CV-AM staining solution was added to the tubes and incubated for 60 min at 37 °C, then 1 μl of 0.5 uM SG staining solution was added and incubated for 15 min at RT. To remove excess amounts of stain, the samples were washed with 7H9-ADC once (for *Mtb* samples fixation was performed using 4% paraformaldehyde/PBS in BSL 3). All samples were diluted with 150 μM NaCl containing 0.05% Tween 80 and analysed by NovoCyte Flow Cytometer [ACEA Biosciences Inc., San Diego, CA, USA] at excitation wavelengths of 488 (SG) nm and 405 (CV-AM) nm. DNase I treated and untreated control, live and HK cells were also prepared^31^.

### Biofilm formation

BCG, *M. intracellulare*, and *M. avium* were grown in a static condition on 200 ml of Sauton medium in L-type culture bottles at 37 °C. In the half of culture, DNase I [Takara, Kusatsu, Shiga, Japan] was added to be 0.25 units/ml every week. Five weeks of static culture allowed exclusive growth of all bacteria as pellicle biofilm on the surface and pellicle biofilms were collected by filtration with 0.45 μm membrane filter and their wet biomass weights were measured.

### Bacterial viability after antibiotics treatment

BCG, *M. intracellulare,* and *M. avium* were grown until an OD_600_ of 0.1 in 7H9/ADC broth. The cultures were then diluted in fresh medium to an OD_600_ of 0.001 with/without DNase I and cultured for a further 72 h at 37 °C on the rotator. After that, bacteria were again diluted in fresh medium to an OD_600_ of 0.001 followed by treatment with INH (0.1 μg/ml), RMP (0.5 μg/ml), AMK (2.5 μg/ml), and CLA (5 μg/ml) for 6 and 24 h; we also compared control samples without antibiotics. After antibiotics treatment, we prepared ten-fold serial dilutions of each sample, from 10^−1^ to 10^−6^, and then plated in triplicate on 7H11-OADC agar. The plates were cultured at 37 °C for 3 weeks. The number of colonies was then counted and CFUs (CFU/ml) were calculated.

### DNA extraction and electrophoresis

Bacterial strains were grown to an OD_600_ of 1.5 in 7H9/ADC broth. This was followed by collection of the BCG, *M. intracellulare, Mtb,* and *M. avium* pellet by centrifuging the samples at 3500 rpm at room temperature (RT) for 20 min. Bacterial pellets were suspended in 200 μl of TE lysis buffer [1 M Tris–HCL (pH 8.0) and EDTA (ethylenediaminetetraacetic acid) in Treff tube and frozen at − 80 °C for about 1 h. Samples were then mixed and washed with chloroform/methanol. All liquid was then removed by centrifugation at 15,000*g* at RT for 10 min, dried at 50 °C for about 1 h until the pellet color became clearer, and resuspended by 200 μl TE with 20 μl of 1 M Tris–HCl pH 9.0 buffers. Next 2.2 μl of 10 mg/mL lysozyme (final 100 μg/ml) was added and samples incubated at 37 °C, overnight. Five μl of 10 mg/ml proteinase K (Invitrogen) in the presence of 10% SDS was added. All samples were incubated at 50 °C for more than 3 h to lyse the cell wall and membrane. gDNA was extracted from each sample by addition of an equal volume of 3 M pH 5.2 sodium acetate and phenol: chloroform: isoamyl alcohol solution (25:24:1 vol/vol/vol) and mixed gently by inverting the tubes for a few minutes. Next, all the samples were centrifuged for 10 min, 15,000*g* at RT, and the supernatant containing gDNA was transferred to a sterilized Eppendorf tube. gDNA was precipitated with isopropanol and was centrifuged for 10 min at 15,000*g*, RT. The supernatant was discarded and pellet washed with 400 μl of 70% ethanol. The pellet was centrifuged for 10 min at 15,000*g*, RT, and the supernatant was decanted gently. After that, RNase A (23 μl of 10 mg/ml; Macherey–Nagel, Germany) was added to DNA samples, diluted with 200 μl TE buffer, and incubated at 37 °C for more than 30 min. An equal volume of 3 M pH 5.2 Sodium Acetate and phenol: chloroform: isoamyl alcohol solution were added and the samples were then centrifuged again for 10 min at 15,000*g*, RT. The supernatant was transferred to a sterilized Eppendorf tube, washed with chilled isopropanol, and then DNA was washed with 70% alcohol. The pellet was air-dried for about 10 min and incubated with TE buffer at 50 °C overnight to dissolve and frozen at − 20 °C for storage.

The same protocol was used for eDNA extraction, but without the bacterial membrane destruction step. Briefly, bacterial pellets were suspended in 200 μl of TE lysis buffer in Treff tube without freezing step. Then, we added to the pellet an equal volume of 3 M sodium acetate pH 5.2 and phenol: chloroform: isoamyl alcohol solution (25:24:1 vol/vol/vol) and mixed gently. eDNA fraction was extracted by centrifugation for 10 min, 15,000*g* at RT. The supernatant contained eDNA was transferred to a sterilized Eppendorf tube and precipitated with isopropanol (10 min, 15,000*g* at RT). eDNA pellet washed with 400 μl of 70% ethanol and diluted in 200 μl TE and incubated with 23 μl of RNase A (10 mg/ml) at 37 °C for more than 30 min. Next, eDNA was extracted again with sodium acetate and phenol: chloroform: isoamyl alcohol solution, precipitated with isopropanol, and washed with 70% ethanol, as described above. The air-dried eDNA pellet was dissolved with TE buffer and store at − 20 °C.

The quality and quantity of the DNA samples were assessed using the gDNA and eDNA extracted from BCG, *M. intracellulare, Mtb*, and *M. avium* and fractionated were visualized by automated electrophoresis Agilent 2200 TapeStation [Agilent Technologies, California, USA] was done using gDNA and eDNA^38^.

### Evaluation of eDNA by staining with PMAxx and real time-PCR

BCG was grown until an OD_600_ of 1.0 in 7H9/ADC broth at 37 °C. Live and dead control samples were prepared (dead cells, heat-killed at 95 °C for 5 min). Four hundred μl aliquots of bacterial culture were added into clear microcentrifuge tubes. Each sample was treated with/without PMAxx at a final concentration of 25 uM (e.g., 1 uL of 10 mM stock in 400 uL). The tubes were then incubated in the darkroom for 10 min at RT and exposed to a light-emitting diode (LED) for 15 min to cross-link PMAxx to DNA. Cells were washed and pelleted by centrifuging at 5000*g* for 10 min and collected. gDNA and eDNA were extracted by phenol: chloroform: isoamyl alcohol solution method described above and samples applied as the templates of RT-PCR targeting 16S rRNA.

### Next-generation sequencing of DNA

eDNA and gDNA samples from bacteria such as BCG, *M. intracellulare, Mtb,* and *M. avium* were sequenced by MiSeq Illumina sequencer.

Sequencing libraries were prepared using Nextera XT DNA Library Prep Kit (Illumina Inc., San Diego, CA, USA) according to the manufacturer’s instructions and purified by AMPure XP beads (Beckman Coulter Inc., Brea, CA, USA). The DNA concentration of each purified library with adapters was measured by Qubit Fluorometer with Qubit dsDNA HS assay kit (Thermo Fisher Scientific Inc., Waltham, MA, USA) and the library size was checked by Agilent 2100 Bioanalyzer with High Sensitivity DNA kit (Agilent Technologies, Santa Clara, CA, USA). Based on the measured DNA concentration and the size, the molarity of each DNA library was calculated and normalized to 4 nM. Each 4 nM DNA library was pooled and sequenced by the MiSeq system with MiSeq Reagent Kit v3 (Illumina) by following the manufacturer’s instructions.

### Comparison of gDNA and eDNA

The genomic sequences and the annotation data of BCG (BCG Tokyo), *M. intracellulare* (ATCC 13,950), *Mtb* (H37Rv), and *M. avium* (104) were obtained from the release 40 of Ensemble Bacteria^37^. The paired-end sequences of gDNA and eDNA samples were filtered using Sam tools^39^ with a maxi parameter of 0.5. The filtered sequences were mapped to each *Mycobacterium* species genome using BWA^40^. The number of reads mapped for each gene was calculated using feature counts^41^. The counts were then normalized by the total read number in a sample, yielding the relative abundance of a gene in a sample. The abundance ratio of eDNA to gDNA was calculated by dividing the relative abundance of eDNA by that of gDNA.

### Statistics

UNIANOVA statistics were performed using IBM SPSS 22.0 software (SPSS, Chicago, IL, USA). Data was analyzed using Games–Howell post-hoc test, Wilcoxon/Kruskal–Wallis non-parametric test, and Student *t*-test. Differences were considered significant when the *P*-value was < 0.05.

## Supplementary Information

Supplementary Information.
