# Supplementary material for: Extracellular DNA of slow growers of mycobacteria and its contribution to biofilm formation and drug tolerance
Source: Sci Rep. 2021 May 26;11:10953. doi: 10.1038/s41598-021-90156-z (PMC8155028; doi:10.1038/s41598-021-90156-z)
Supplement: Supplementary file 1 — Supplementary Information. [file 41598_2021_90156_MOESM1_ESM.docx]

**Supplementary Data**

**Extracellular DNA of** **slow growers of mycobacteria and its contribution to biofilm formation and drug tolerance**

Aleksandr Ilinov, Akihito Nishiyama, Hiroki Namba, Yukari Fukushima, Hayato Takihara, Chie Nakajima, Anna Savitskaya, Gebremichal Gebretsadik, Mariko Hakamata, Yuriko Ozeki, Yoshitaka Tateishi, Shujiro Okuda, Yasuhiko Suzuki, Yuri S. Vinnik, and Sohkichi Matsumoto.

| **MAV number** | **Ratio g/eDNAs** | **MAV number** | **Ratio g/eDNAs** | **MAV number** | **Ratio g/eDNAs** |
| --- | --- | --- | --- | --- | --- |
| MAV_0773 | 0,984088582 | MAV_0801 | 1,912619484 | MAV_0827 | 2,135435716 |
| MAV_0774 | 0,947922809 | MAV_0802 | 1,971412523 | MAV_0828 | 2,027953366 |
| MAV_0775 | 1,042151562 | MAV_0803 | 2,209791402 | MAV_0829 | 1,929270009 |
| MAV_0778 | 1,024438455 | MAV_0804 | 1,973073486 | MAV_0830 | 1,697504564 |
| MAV_0779 | 1,6820398 | MAV_0805 | 2,056173193 | MAV_0831 | 1,692142141 |
| MAV_0780 | 1,962161511 | MAV_0806 | 2,386102672 | MAV_0832 | 1,5295878 |
| MAV_0781 | 1,875089435 | MAV_0807 | 2,316083051 | MAV_0833 | 1,676953661 |
| MAV_0782 | 1,493055073 | MAV_0808 | 2,572335075 | MAV_0834 | 1,96047605 |
| MAV_0783 | 1,465399357 | MAV_0809 | 2,75890341 | MAV_0835 | 1,9824219 |
| MAV_0784 | 1,472820286 | MAV_0810 | 2,410880268 | MAV_0836 | 1,736244738 |
| MAV_0785 | 1,518264951 | MAV_0811 | 2,487765614 | MAV_0837 | 1,636132279 |
| MAV_0786 | 1,876885429 | MAV_0811 | 2,828544401 | MAV_0838 | 1,529603423 |
| MAV_0787 | 1,651494006 | MAV_0812 | 2,91917224 | MAV_0839 | 1,718625091 |
| MAV_0788 | 1,635627469 | MAV_0813 | 2,797255561 | MAV_0840 | 1,609020515 |
| MAV_0789 | 1,714988937 | MAV_0814 | 2,32226046 | MAV_0841 | 1,72277625 |
| MAV_0790 | 1,713648129 | MAV_0815 | 3,174553989 | MAV_0842 | 1,630849304 |
| MAV_0791 | 1,960290668 | MAV_0816 | 2,731180527 | MAV_0843 | 0,903866066 |
| MAV_0792 | 2,109314762 | MAV_0817 | 2,22184754 | MAV_0844 | 0,948717284 |
| MAV_0793 | 2,325025457 | MAV_0818 | 1,730652135 | MAV_0846 | 1,003070879 |
| MAV_0794 | 2,309457619 | MAV_0819 | 2,056834436 | MAV_0848 | 0,982212431 |
| MAV_0795 | 2,179489113 | MAV_0820 | 2,191953945 | MAV_0849 | 0,923757295 |
| MAV_0796 | 2,029224707 | MAV_0822 | 2,016597629 | MAV_0850 | 0,889624274 |
| MAV_0797 | 1,625478607 | MAV_0823 | 2,104970568 | MAV_0852 | 1,188577086 |
| MAV_0798 | 1,88557973 | MAV_0824 | 2,030279726 | MAV_0853 | 1,044704604 |
| MAV_0799 | 1,723516902 | MAV_0825 | 1,738446232 | MAV_0854 | 1,007391799 |
| MAV_0800 | 1,995020165 | MAV_0826 | 1,894991782 |  |  |

**Supplementary Table S1.** Genes list, including accumulated genes in eDNA of *M. avium* and their ratio of eDNA/gDNA.

| **16S** | **Exp. 1** | **Exp. 2** | **Average** |
| --- | --- | --- | --- |
| **gDNA** | 1.76 | 1.21 | 1.5 |
| **eDNA** | 3.09 | 4.56 | 3.83 |

**Supplementary Table S2. The fold-ratio of amplification of 16S by qPCR**

To verify the method of extraction of eDNA, we stained BCG or not with a photo-reactive DNA-binding dye - PMAxx at RT for 10 minutes and exposed to a light-emitting diode (LED) for 15 minutes. gDNA and eDNA were then extracted as the protocol described in the method section and qPCR was performed targeting 16S rDNA of BCG. Experiments were independently repeated twice (Exp. 1 and 2) and average amplification ratio of PMAxx untreated/exposed samples is calculated.

**Supplementary Figure S1. Differential effect of DNase I treatment on drug susceptibility of *M. intracellulare* and *M. avium*.**

*M. intracellulare* and *M. avium* were – untreated (-DNase I) or – treated (+DNase I) at 37 °C for 72 hours and further incubated with AMK, CLA or not, for 6 and 24 hours. CFU/ml was determined and normalized with that of control samples. The average viability ratio of each sample (mean ± SD, n = 3) is indicated. *, *p*<0.05.

**
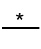
**


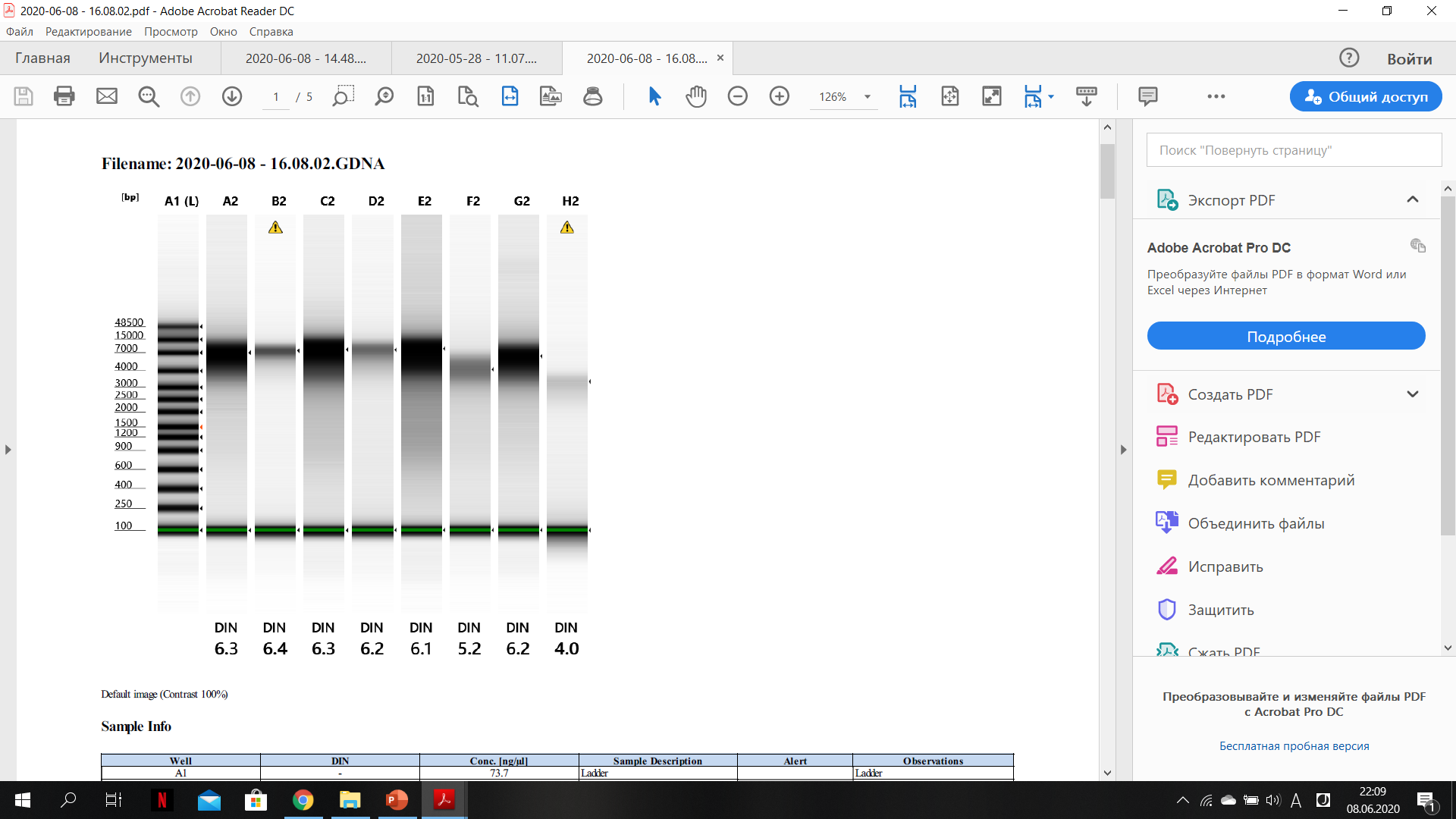


***M. intracellulare***

**[bp]**

**A1 (L)**

**A2**

**B2**

**C2**

**D2**

**E2**

**F2**

**G2**

**H2**

48500

15000

7000

4000

3000

2500

2000

1500

1200

400

900

100

250

600

**gDNA**

**gDNA**

**gDNA**

**eDNA**

**eDNA**

**eDNA**

**eDNA**

**gDNA**

***M. avium***

**BCG**

***Mtb***

4000

7000


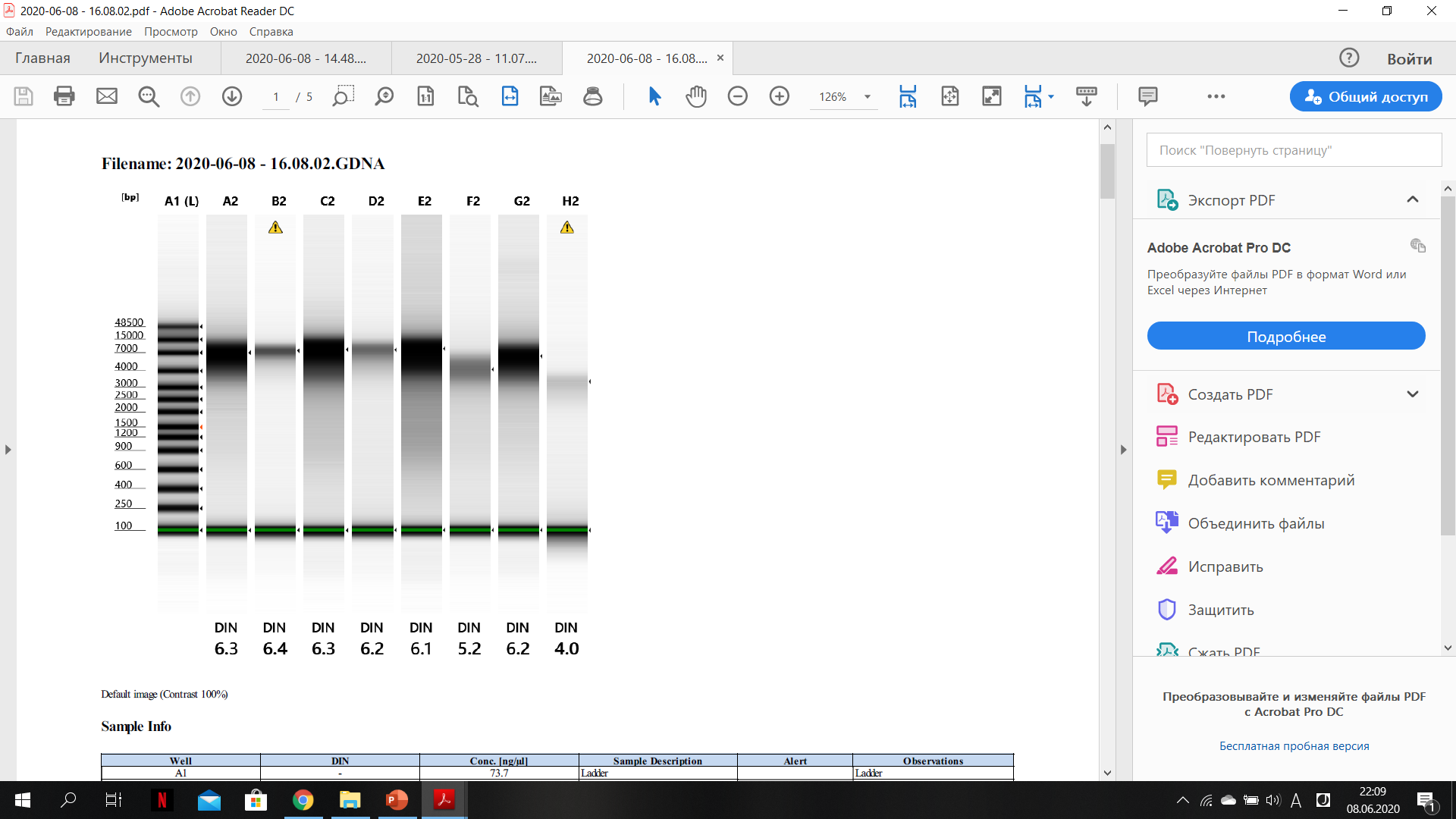

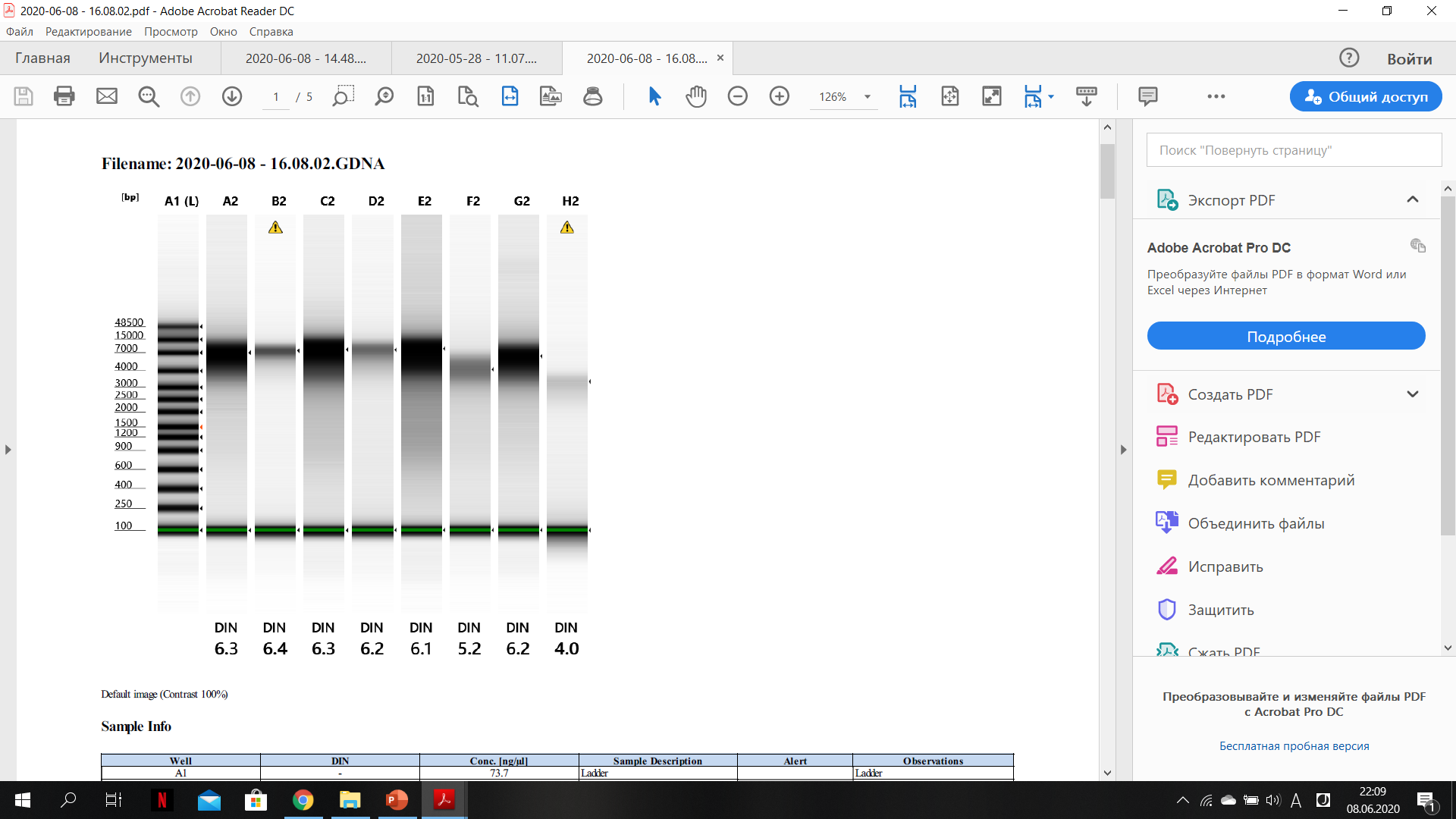


**Supplementary Figure S2. Gel electrophoresis of genomic DNA (gDNA) and eDNA of mycobacteria.**

gDNA and eDNA extracted from BCG, *M. intracellulare, Mtb*, and *M. avium* and fractionated were visualized by automated electrophoresis Agilent 2200 TapeStation. Standards of molecular weight are also loaded. White lines indicate where parts from the same gel were merged.


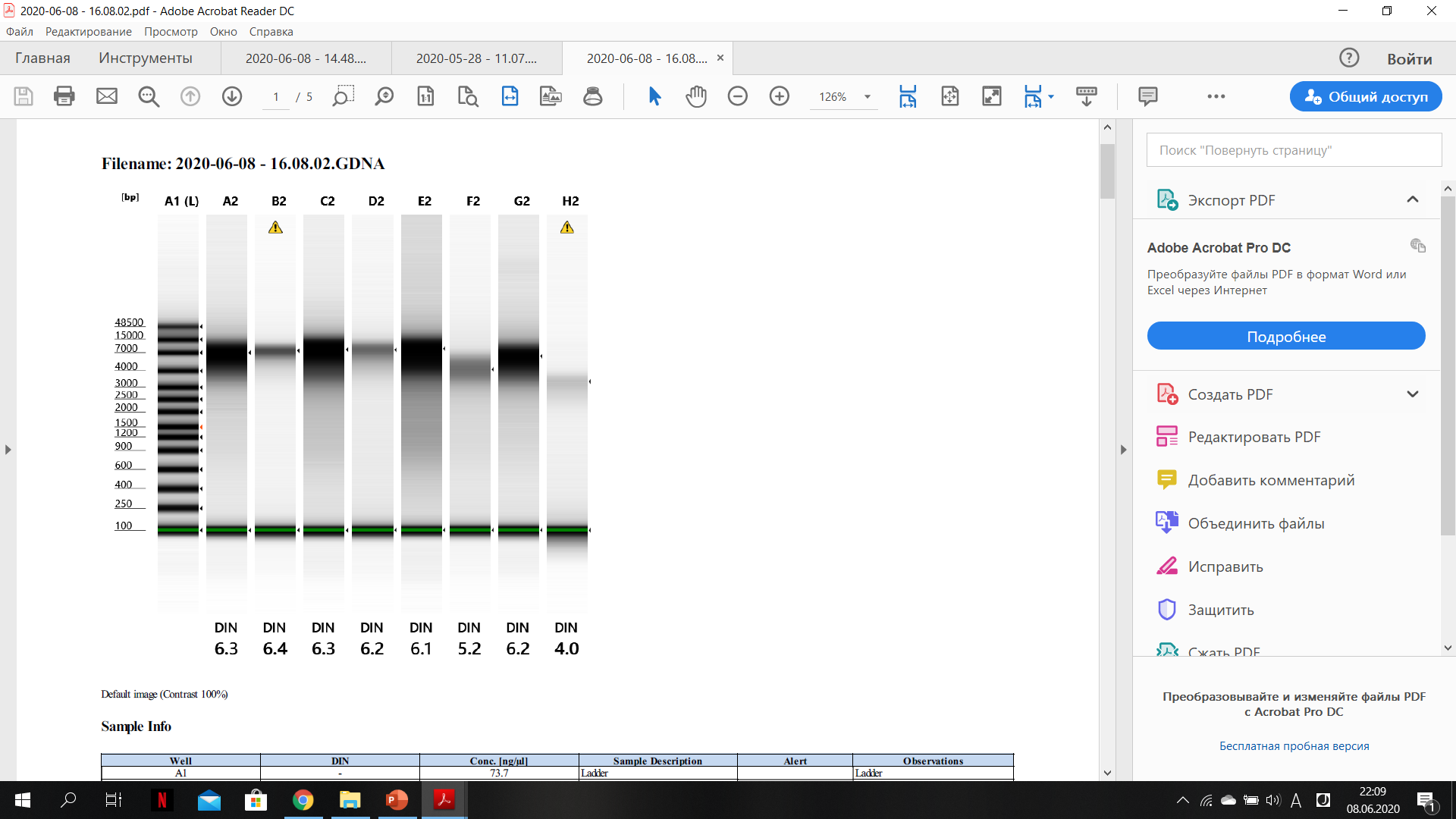


**Supplementary Figure S3. Original gel electrophoresis of genomic DNA (gDNA) and eDNA of mycobacteria.**
